# Investigating the role of G-quadruplexes at *Saccharomyces cerevisiae* telomeres

**DOI:** 10.15698/mic2022.06.778

**Published:** 2022-05-19

**Authors:** Sonia Stinus, Fernando R. Rosas Bringas, Lisa Wanders, Michael Chang

**Affiliations:** 1 European Research Institute for the Biology of Ageing, University of Groningen, University Medical Center Groningen, Groningen, The Netherlands

**Keywords:** telomere, G-quadruplex, budding yeast, tlc1-tm, Cdc13, Pif1, Saccharomycotina

## Abstract

The G-quadruplex consensus motif G_≥3_N_x_G_≥3_N_x_G_≥3_N_x_G_≥3_ is found at telomeres of many species, ranging from yeast to plants to humans, but the biological significance of this fact remains largely unknown. In this study, we examine the in vivo relevance of telomeric G-quadruplexes in the budding yeast *Saccharomyces cerevisiae* by expressing a mutant telomerase RNA subunit (tlc1-tm) that introduces mutant [(TG)_0–4_TGG]_x_ATTTGG telomeric repeats instead of wild-type (TG)_0-6_TGGGTGTG(G)_0-1_ repeats to the distal ends of telomeres. The *tlc1-tm* telomere sequences lack the GGG motif present in every wild-type repeat and, therefore, are expected to be impaired in the formation of G-quadruplexes. Circular dichroism analysis of oligonucleotides consisting of *tlc1-tm* telomeric sequence is consistent with this hypothesis. We have previously shown that *tlc1-tm* cells grow similarly to wild-type cells, suggesting that the ability to form telomeric G-quadruplexes is not essential for telomere capping in *S. cerevisiae* cells.

## INTRODUCTION

The physical ends of eukaryotic chromosomes are protected by nucleoprotein complexes known as telomeres. Telomeres protect chromosome ends from degradation, from telomere-telomere fusion events, and from being recognized as double-stranded DNA breaks [1]. In most eukaryotic species, telomeres consist of double-stranded G/C-rich DNA followed by a G-rich 3′ single-stranded overhang. Proper telomere function is ensured by the specialized proteins bound to the double-stranded and single-stranded telomeric repeats. Telomere length is kept in a state of dynamic equilibrium. Incomplete DNA replication and nucleolytic degradation cause telomeres to shorten, while the reverse transcriptase telomerase is responsible for telomere lengthening [1]. Telomerase extends the 3′ overhang of telomeres by iterative reverse transcription using its RNA subunit as a template.

Due to the G-rich nature of the telomeric repeats, telomeric DNA has the potential to form G-quadruplexes, which are highly stable secondary structures composed of Hoogsteen hydrogen-bonded guanines arranged in planar G-tetrads stacked together [2]. Intramolecular G-quadruplexes are predicted to form within sequences containing four runs of at least three guanines (G_≥3_N_x_G_≥3_N_x_G_≥3_N_x_G_≥3_), and the telomeric DNA of most eukaryotic organisms conform to this consensus sequence. While most studies on G-quadruplexes have been carried out in vitro, there is also in vivo work supporting the existence of G-quadruplexes at telomeres. The most direct evidence comes from studies in ciliates. The telomere-binding protein TEBPβ, from the related ciliates *Oxytricha nova* and *Stylonychia lemnae*, can promote the formation of G-quadruplexes in vitro [3, 4]. Knockdown of TEBPβ in *S. lemnae* eliminates detection of telomeric G-quadruplexes in vivo using the Sty3 G-quadruplex antibody in nuclear staining experiments [4]. Telomeric G-quadruplexes are not detected during S phase, presumably to allow replication of telomeres [4]. Unfolding of telomeric G-quadruplexes during S phase requires phosphorylation of TEBPβ, as well as telomerase and a RecQ-like helicase [4–6].

In the budding yeast *Saccharomyces cerevisiae*, the main telomere binding protein Rap1, like TEBPβ, can bind and promote the formation of G-quadruplexes in vitro [7, 8]. In contrast to the findings in ciliates, chromatin immunoprecipitation experiments using the BG4 G-quadruplex antibody suggest that telomeric G-quadruplexes may form in late S phase, when *S. cerevisiae* 3′ overhangs reach their longest length [9]. The telomerase subunit Est1 can also promote G-quadruplex formation in vitro, and cells expressing Est1 mutants deficient in this activity exhibit gradual telomere shortening and replicative senescence, suggesting a potential positive role for G-quadruplexes in telomerase-mediated extension of telomeres [10]. In addition, there is evidence to suggest that stabilization of G-quadruplexes suppresses the temperature sensitivity of the telomere capping-defective *cdc13-1* mutant [11]. Cdc13 is a single-stranded telomeric DNA binding protein; the *cdc13-1* mutant loses the ability to block excessive nucleolytic resection of telomeric DNA at elevated temperatures, resulting in an accumulation of single-stranded telomeric DNA [12, 13]. The folding of this DNA into G-quadruplexes has been proposed to facilitate telomere capping by inhibiting further nucleolytic resection [11]. Despite these findings, it remains unclear whether G-quadruplexes have an evolutionarily conserved function in telomere biology [14].

In this study, we examined the function of G-quadruplexes at *S. cerevisiae* telomeres by expressing a mutant telomerase RNA subunit (tlc1-tm) that introduces [(TG)_0–4_TGG]_x_ATTTGG mutant telomeric repeats instead of wild-type (TG)_0-6_TGGGTGTG(G)_0-1_ repeats [15, 16]. The mutant repeats are impaired in the formation of G-quadruplexes, and we have previously shown that *tlc1-tm* repeats are poorly bound by Rap1 [17]. Despite being deficient in telomeric G-quadruplex formation, *tlc1-tm* cells are viable and grow as well as wild-type cells, suggesting that the ability to form telomeric G-quadruplexes is not essential for telomere capping and cell viability in *S. cerevisiae*.

## RESULTS

### *tlc1-tm* mutant telomere sequences have reduced potential to form G-quadruplexes

To assess the role of G-quadruplexes at yeast telomeres, we require a yeast strain with telomeric DNA sequences that lack the potential to form G-quadruplexes. Such a strain can be obtained by mutating the template sequence of the RNA subunit of telomerase, TLC1. The vast majority of mutations to the TLC1 template sequence causes disruption of telomerase enzymatic activity, and consequently, replicative senescence [18]. Those that do not are often associated with slow growth, dramatic alterations in telomere profile (i.e. elongated, very short, or extensively degraded), and aberrant chromosome separation and segregation [18, 19]. The *tlc1-tm* mutant introduces [(TG)_0–4_TGG]_x_ATTTGG mutant telomeric repeats instead of wild-type (TG)_0-6_TGGGTGTG(G)_0–1_ repeats, and grows similar to a wild-type strain, even when one telomere consists entirely of mutant sequence [15–17]. Telomeres in the *tlc1-tm* mutant are on average longer and more heterogeneous in length than in wild-type strains [17], but the telomere profile of *tlc1-tm* is much less dramatically altered compared to most other mutants of *TLC1* with altered template sequences [18, 19].

**FIGURE 1:**
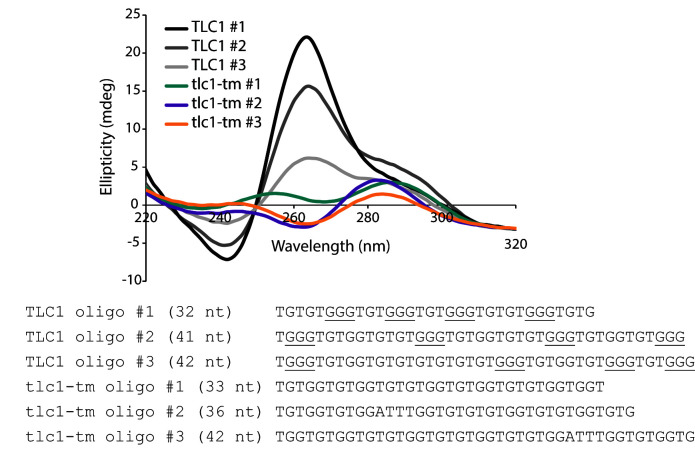
FIGURE 1: *tlc1-tm* mutant telomere sequences are impaired in forming G-quadruplexes. CD spectra of oligonucleotides with either wild-type or *tlc1-tm* telomeric sequence. Average of three measurements is plotted.

The lack of the GGG motif in the mutant repeat sequence should weaken the potential of G-quadruplex formation. To test this idea, we used the G-quadruplex prediction tool, G4Hunter, where a score greater than 1.2 indicates high G-quadruplex-forming potential [20]. While analysis of wild-type sequences gave G4Hunter scores of 1.366, 1.375, and 1.286 (see sequences used in Figure 1), none of the three analyzed mutant *tlc1-tm* sequences has a score greater than 1, thus indicating that the mutant telomeric sequences have reduced G-quadruplex-forming potential. To validate this hypothesis, we subjected oligonucleotides with either wild-type or *tlc1-tm* telomere sequences to circular dichroism (CD) analysis after incubation with potassium. In agreement with previous studies reporting that yeast telomeric DNA can fold into G-quadruplex structures in vitro [7, 21], we find that all three oligonucleotides composed of wild-type telomeric sequence generate a negative peak at 240 nm and a positive peak at 263 nm (Figure 1), which is a pattern consistent with parallel G-quadruplex formation. In contrast, none of the oligonucleotides with *tlc1-tm* telomere sequence form such a pattern (Figure 1). It is formally possible that *tlc1-tm* telomeres form less stable two-quartet G-quadruplexes (which have a consensus sequence of G_≥2_N_x_G_≥2_N_x_G_≥2_N_x_G_≥2_). Indeed, the spectra of tlc1-tm oligonucleotides #2 and #3, despite having low amplitude, could indicate an antiparallel G-quadruplex structure, which is characterized by a negative peak near 260 nm and positive ones at 240 and 295 nm. Nevertheless, our findings indicate that the formation of any G-quadruplex structures by wild-type telomeric sequence should be, at minimum, greatly perturbed in *tlc1-tm* telomeric sequence.

### Deletion of *PIF1* suppresses *cdc13-1*, but not *cdc13-1 tlc1-tm*, temperature sensitivity

**FIGURE 2:**
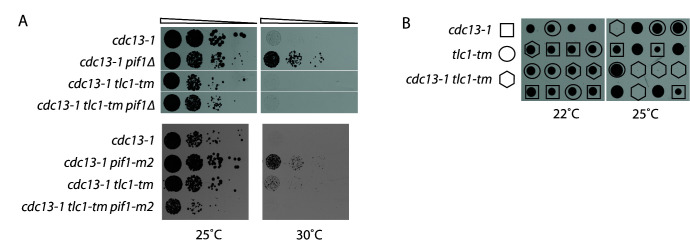
FIGURE 2: Deletion of *PIF1* does not rescue telomere capping-deficient *tlc1-tm* cells. **(A)** Tenfold serial dilutions of strains with the indicated genotypes were spotted on YPD plates and grown at 25ºC or 30ºC. **(B)** A *cdc13-1/CDC13 tlc1-tm/TLC1* diploid strain was sporulated and the resulting tetrads were dissected on YPD plates, which were incubated at 22ºC or 25ºC. Each column of colonies arose from a single tetrad.

To test whether *tlc1-tm* telomere sequences are defective in forming G-quadruplexes in vivo, we stabilized G-quadruplexes in the telomere capping-defective *cdc13-1* mutant by deleting PIF1. Pif1 is a helicase and a potent unwinder of G-quadruplexes [22]. Suppression of *cdc13-1* temperature sensitivity by *pif1Δ* has already been reported [23]. We find that *pif1Δ* cannot suppress the temperature sensitivity of *cdc13-1* in a *tlc1-tm* background (Figure 2A). We observe the same effect when using the *pif1-m2* allele, which is specifically deficient for the nuclear isoform of Pif1 [24]. Thus, *tlc1-tm* telomeres remain uncapped even in the absence of Pif1, possibly due to a lack of G-quadruplexes to stabilize.

We noticed that *cdc13-1 tlc1-tm* cells grow more slowly than *cdc13-1* cells even at 25ºC (Figure 2A; top panel). This effect is even more striking upon dissection of a *cdc13-1/CDC13 tlc1-tm/TLC1* diploid. We find no difference in the colony size formed by the haploid progeny at 22ºC, regardless of their *CDC13* and *TLC1* status (Figure 2B). However, *cdc13-1 tlc1-tm* spores were unable to germinate at 25ºC (Figure 2B), although the *cdc13-1 tlc1-tm* spores that germinated at 22ºC were able to grow at 25ºC (Figure 2A). These findings suggest that G-quadruplex-mediated capping may be important even at a temperature (25ºC) where the *Cdc13-1* mutant protein is only modestly impaired [25].

While our findings are consistent with a previously proposed model in which G-quadruplexes protect *cdc13-1* telomeres [11], the effect of *tlc1-tm* on *cdc13-1* cells may instead be due to reduced levels of Rap1 at *tlc1-tm* telomeres [17] rather than a disruption in G-quadruplex formation. However, we do not favor this possibility because telomeres in *tlc1-tm* cells still retain wild-type telomeric sequence in their centromere-proximal regions, so that telomere-bound Rap1 is only reduced by 40% [17].

## DISCUSSION

In this study, we investigated the function of G-quadruplexes at *S. cerevisiae* telomeres using the *tlc1-tm* mutant, which causes the addition of mutant telomeric repeats that are defective in forming G-quadruplexes. Our findings suggest that G-quadruplex formation at telomeres is not essential for telomere capping nor cell viability in *S. cerevisiae*. In addition, our findings are not consistent with a previously proposed model whereby Est1-mediated G-quadruplex formation is required for telomerase activity [10], since *tlc1-tm* telomeres are efficiently extended by telomerase [17]. While we cannot exclude the possibility that less stable G-quadruplex structures (e.g. two-quartet G-quadruplexes) are able to form at *tlc1-tm* telomeres, there are other viable *tlc1* template mutants that result in telomeric repeats that lack even a double GG motif [18, 19]. Nonetheless, our findings are in agreement with a previously proposed model suggesting that telomeric G-quadruplexes serve as capping structures to protect *cdc13-1* telomeres [11], and it is also possible that telomeric G-quadruplexes are important for telomere function when *S. cerevisiae* cells are grown in stress-inducing conditions. Furthermore, we have previously reported several telomeric defects (e.g. disruption of telomere length homeostasis) in *tlc1-tm* cells [17]. While we believe that most of these defects can be largely attributed to depletion of telomere-bound Rap1, it is formally possible that impairment in the formation of telomeric G-quadruplexes could contribute to some of these defects.

The telomere repeats of *S. cerevisiae* and other Saccharomycotina species are highly divergent and differ from the TTAGGG or TTAGGG-like repeats found in many other eukaryotic species [26, 27]. Budding yeast repeats can be quite long, occasionally degenerate, and often non-G/C-rich [28, 29]. Many of the budding yeast telomere sequences do not conform to the G_≥3_N_x_G_≥3_N_x_G_≥3_N_x_G_≥3_ G-quadruplex consensus. Changes in the sequence of the telomeric repeats were accompanied by co-evolution of telomere-binding proteins. In organisms with TTAGGG telomeric repeats, the double-stranded telomeric sequence is typically recognized by proteins homologous to mammalian TRF1 and TRF2, while the single-stranded telomeric sequence is bound by proteins homologous to mammalian POT1. Telomere association of these proteins is highly sequence specific [30, 31], so mutating the template region of telomerase RNA leads to a loss of cell viability [32–34]. In contrast, the telomeres of Saccharomycotina budding yeast species (with the exception of the Yarrowia clade, one of the basal lineages of Saccharomycotina [35]) are bound by Rap1 and Cdc13. Rap1 and Cdc13 have the possibility to accommodate different target sequences, thereby facilitating the rapid evolution of budding yeast telomeric sequences [29]. A consequence of this rapid evolution may be the loss of a need for telomeric G-quadruplexes. Further studies are needed to determine whether G-quadruplexes are required for proper telomere maintenance in species with TTAGGG telomeric repeats. One recent study has reported that folding of telomeric DNA newly synthesized by human telomerase into G-quadruplexes is important to support telomerase function, which the authors suggest could provide an explanation for the evolutionary conservation of the G-quadruplex-forming potential of telomeric sequence [36]. Addressing this question is especially relevant given that G-quadruplexes have increasingly been proposed as therapeutic targets in oncology [37].

If G-quadruplexes are not essential for telomere capping in *S. cerevisiae*, why does Rap1 have the ability to bind and promote the formation of G-quadruplexes [7, 8]? We propose two possible explanations. First, this ability may have been required for telomere capping, but this requirement was lost during the evolution of the Saccharomycotina subdivision. Rudimentary G-quadruplex-based capping in *cdc13-1* mutants [11] may be an evolutionary remnant of this requirement, so it would be interesting to test whether suppression of *cdc13-1* capping defects by G-quadruplex-stabilizing treatments is dependent on Rap1. Second, the ability of Rap1 to bind and promote the formation of G-quadruplexes may be important for Rap1’s function as a transcriptional regulator [38], rather than for telomere capping. Consistent with this hypothesis, G-quadruplex-forming sequences are strongly enriched at promoters and are thought to influence transcription [39]. These two hypotheses are not mutually exclusive, and it will be interesting to explore their validity in future studies.

## MATERIALS AND METHODS

### Yeast strains

Standard yeast media and growth conditions were used [40, 41]. Yeast strains used in this study are listed in Table 1. Deletion of *PIF1* was accomplished by PCR-based gene deletion [42]. Knock-in of the *tlc1-tm* allele was accomplished by PCR amplification of *tlc1-tm* from either MCY415 or MCY416, using primers oSMS1 (5′-ACCTGCCTTTGCAGATCCTT-3′) and TLC1-RV (5′-TTATCTTTGGTTCCTTGCCG-3′), followed by transformation of the PCR product into yeast cells using the LiAc-based method [43]. The diploid strain dissected in Figure 2B was generated by knock-in of the *tlc1-tm* allele into SSY238. The spore colonies were genotyped by replica plating onto YPD + clonNAT plates (to select for the *tlc1-tm* allele) and YPD plates that were subsequently incubated at 30ºC (to identify *cdc13-1* spore colonies, which do not grow at 30ºC).

**TABLE 1.** Yeast strains used in this study.

**Strain name**	**Strain background**	**Mating type**	**Genotype**	**Source**
YBJ120	PSY316	alpha	*cdc13-1 ura3-52 leu2-3,112 his3-200 ade2-101 lys2-801*	Brad Johnson
SSY156	PSY316	alpha	*cdc13-1tlc1-tm::kanMX ura3-52 leu2-3,112 his3-200 ade2-101 lys2-801*	This study
SSY228	PSY316	alpha	*cdc13-1 pif1ΔnatMX ura3-52 leu2-3,112 his3-200 ade2-101 lys2-801*	This study
SSY230	PSY316	alpha	*cdc13-1 pif1ΔnatMX tlc1-tm::kanMX ura3-52 leu2-3,112 his3-200 ade2-101 lys2-801*	This study
SSY238	W303	a/alpha	*cdc13-1/CDC13 ade2-1/ade2-1 can1-100/can1-100 his3-11,15/his3-11,15 leu2-3,112/leu2-3,112 trp1-1/trp1-1 ura3-1/ura3-1 RAD5/RAD5*	This study
SSY279	W303	a	*cdc13-1 ade2-1 can1-100 leu2-3,112 his3-11,15 trp1-1 ura3-1*	This study
SSY280	W303	a	*cdc13-1 pif1-m2 ade2-1 can1-100 leu2-3,112 his3-11,15 trp1-1 ura3-1*	This study
SSY281	W303	a	*cdc13-1tlc1-tm::natMX ade2-1 can1-100 leu2-3,112 his3-11,15 trp1-1 ura3-1*	This study
SSY282	W303	a	*cdc13-1tlc1-tm::natMX pif1-m2 ade2-1 can1-100 leu2-3,112 his3-11,15 trp1-1 ura3-1*	This study
MCY415	BY4742	alpha	*tlc1-tm::kanMX his3Δ1 leu2Δ0 ura3Δ0*	[44]
MCY416	BY4742	alpha	*tlc1-tm::natMX his3Δ1 leu2Δ0 ura3Δ0*	[15]

### Spot assays

Cultures for spot assays were grown overnight and diluted to an optical density (OD_600_) of 0.5, from which four serial 1:10 dilutions were spotted onto YPD plates. Plates were incubated at indicated temperatures for 2 or 3 days.

### CD spectroscopy

Oligonucleotides were dissolved in a 10 mM Tris-HCl pH 7.5 and 100 mM KCl solution in a final concentration of 5 μM. The mix was boiled for 5 min at 95ºC and then cooled down overnight. The CD spectra were then measured using a Jasco J-815 spectropolarimeter. Three reads per sample were taken at a wavelength range of 215-350 nm in a quartz cuvette with a 1 cm path length. Data were analyzed using Spekwin32 software.

## Abbreviations:

tlc1-tm: – mutant telomerase RNA subunit
CD: – circular dichroism
